# Environmental complexity positively impacts affective states of broiler chickens

**DOI:** 10.1038/s41598-021-95280-4

**Published:** 2021-08-20

**Authors:** M. G. Anderson, A. M. Campbell, A. Crump, G. Arnott, L. Jacobs

**Affiliations:** 1 Department of Animal and Poultry Sciences, Virginia Polytechnic Institute and State University, Blacksburg, VA USA; 2 Centre for Philosophy of Natural and Social Science, London School of Economics and Political Science, London, UK; 3 School of Biological Sciences, Queen’s University Belfast, Belfast, UK

**Keywords:** Animal behaviour, Emotion, Learning and memory, Motivation

## Abstract

Affective state can bias an animal’s judgement. Animals in positive affective states can interpret ambiguous cues more positively (“optimistically”) than animals in negative affective states. Thus, judgement bias tests can determine an animal’s affective state through their responses to ambiguous cues. We tested the effects of environmental complexity and stocking density on affective states of broiler chickens through a multimodal judgement bias test. Broilers were trained to approach reinforced locations signaled by one color and not to approach unreinforced locations signaled by a different color. Trained birds were tested for latencies to approach three ambiguous cues of intermediate color and location. Broilers discriminated between cues, with shorter latencies to approach ambiguous cues closest to the reinforced cue than cues closest to the unreinforced cue, validating the use of the test in this context. Broilers housed in high-complexity pens approached ambiguous cues faster than birds in low-complexity pens–an optimistic judgement bias, suggesting the former were in a more positive affective state. Broilers from high-density pens tended to approach all cues faster than birds from low-density pens, possibly because resource competition in their home pen increased food motivation. Overall, our study suggests that environmental complexity improves broilers’ affective states, implying animal welfare benefits of environmental enrichment.

## Introduction

Broiler chickens are typically housed in barren environments and at high stocking densities in order to minimize production cost, which has the potential to compromise broiler welfare^1–4^. In conventional housing systems, broilers spend approximately 80% of their time budget lying down, and a positive association between time spent lying down and lameness has been found, which negatively influences broiler welfare^5^. Environmental enrichment (complexity) has a positive impact on animals’ biological functioning and behavior^1,4,6–10^. Broilers with access to elevated platforms experienced improved gait through an increased occurrence of low (good) gait scores, lower (better) flock mean gait score, and lower occurrence of tibial dyschondroplasia compared to broilers without access to platforms^11^. Stocking density is another important environmental factor that can impact broiler chicken welfare aspects, such as leg health, level of bruising and scratches, lameness, and behavioral suppression^12–14^. While housing broilers at high stocking densities maximizes profit for the producer, it has the potential to compromise bird health and welfare as seen through decreased final body weight and feed conversion ratio, as well as increased occurrence of footpad dermatitis and mortality^12,15–17^. Furthermore, high stocking densities reduce space use and therefore activity level, and increase disturbances that lead to decreased plumage and carcass quality^2,16,18–20^. These and other studies show that conventional housing of broiler chickens has negative effects on bird health and welfare, although their effects on affective state are unknown.

The ability to perform highly motivated behaviors is important for good animal welfare^1,21–23^. Three species-specific behaviors have been identified for broilers, whose deprivation may cause negative affective states: perching, dustbathing, and foraging^23–27^. Perching is a natural behavior for jungle fowl (broilers’ ancestors), which seek elevated resting spaces possibly to avoid predation while sleeping^28,29^. Broilers have maintained motivation to perch when given easily accessible perches^30^. When provided perches at a low stocking density, broilers perched for longer (nearly 25% of the observation periods) and were less aggressive than control birds^10^. Dustbathing is another highly-motivated behavior for broilers, as jungle fowl who are deprived of substrate to dustbathe in will exhibit excessive, compensatory dustbathing behavior when eventually provided with a substrate or even dustbathe on wire flooring, highlighting the need to perform this behavior regardless of access to a suitable substrate^31^. Broiler chickens dustbathe throughout their life, even chicks have been shown to dustbathe during the first week of life^32,33^. Furthermore, jungle fowl and broilers have retained the motivation to forage. Even when regularly fed, semi-wild junglefowl foraged for 61% of the time they spent active, as well as spent 34% of their time scratching, a behavior considered to be associated with foraging^34^. Without access to a preferred substrate or environment, domestic fowl forage in feces^35,36^, suggesting that this behavior is highly motivated. Commercial broilers can be deprived of all three of these high-motivation behaviors; they do not have access to perches or proper substrate to dustbathe and forage in. While they do have access to litter, providing litter alone, either clean or reused depending on industry standards, is not enough to stimulate all natural behaviors^30,32,37,38^.

Life experiences, including environmental conditions, can elicit short-term emotions which are defined as functional states elicited by reward or punishment (stimuli that animals work to either gain or avoid)^39^. These emotions are adaptive and help animals appropriately respond to changes in their environment^40^. Emotional responses shape an animal’s mood, which can be defined as long-term, diffuse states that reflect the cumulative valence of emotions over time^41^. Both emotion and mood contribute to an animal’s affective state, which can be measured along a spectrum between positive and negative (valence)^41^. An animal that experiences more positive than negative emotions throughout life, for instance induced by the ability to express play behaviors, will be in an overall positive affective state, compared to animals that have more negative experiences^40^. When an animal experiences more negative than positive emotions, such as chronic or excessive fear and anxiety, the animal will be in an overall negative affective state^6,42,43^. Typically, negative experiences tend to have a stronger influence on affective state than positive experiences^15,44,45^. In order to achieve good welfare, the induction of positive experience must be considered in addition to preventing negative experiences. However, most published studies for agricultural species focus on avoidance of negative affective states such as level of fear^7,46^, or physiological measures^20^, like lameness^8,47^.

Affective states are closely associated with cognition–the mechanisms by which animals acquire, process, store, and act on information from their environment^40^. Affective states influence cognitive processing and cognitive processing impacts affective states^48–50^. When emotions and affective states impact aspects of cognition, such as judgement, attention, and memory, we call this “cognitive bias”^51–53^. Cognitive biases can be used as an indicator of animal welfare^40,52,54–56^.

Humans experiencing a negative affective state (depression, anxiety) tend to interpret ambiguous events negatively and have a pessimistic outlook, whereas humans who are in a more positive affective state tend to interpret the same ambiguous events positively and have a more optimistic outlook^57–60^. Animal responses to ambiguous situations can be quantified using a judgement bias test, assessing cognitive bias. Judgement bias testing is used to determine levels of optimism and pessimism of subjects based on responses to ambiguous cues during testing. Judgement bias cues can be spatial^61^, visual^62^, auditory^63^, olfactory^64^, tactile^65^ or a combination of these (multimodal^54^). Shorter latencies to approach ambiguous cues would indicate optimism, whereas longer latencies to approach ambiguous cues would indicate pessimism^9,40,52,66–70^. A meta-analysis of 71 judgement bias studies on 22 species showed optimistic and pessimistic responses to ambiguous situations resulting from positive or negative affective states, respectively^71^. Thus, judgement bias is considered the “gold standard” for evaluating affective states in animals^72^.

One previous study has evaluated broiler chicken affective state through a judgement bias test. Iyasere et al.^73^ trained broilers on a spatial, go/no-go judgement bias task to discriminate between a reward- (mealworms) and punishment-associated (air puff) cone. Following discrimination, birds treated with corticosterone had longer latencies to displace cones at all cue locations compared to control birds, suggesting a pessimistic bias. A similar test approach was conducted on laying hens, where ‘exploratory’ layer hens (categorized based on novel object and open area test responses) housed with enrichments showed more optimistic responses than exploratory hens housed without enrichments^74^. Therefore, judgement bias tests could be a valuable tool to assess positive affective states in broilers housed under varying environmental conditions. Including an evaluation of lameness in conjunction with judgement bias testing is warranted, as birds were required to walk in judgement bias training and testing.

In the present study, we used a judgement bias test to assess the effect of environmental complexity and stocking density, manipulated in a factorial experiment, on broilers’ affective states. Gait was quantified as a potential confounding factor for the judgement bias test. We hypothesized that birds housed in high-complexity (HC), low-density (LD) environments would respond more optimistically, indicating more positive affective states, compared to birds from low-complexity (LC), high-density (HD) environments. Birds housed in LC/LD or HC/HD environments were predicted to show intermediate levels of optimism.

## Results

### Judgement bias test

Training for the judgement bias task took between 3 and 10 sessions (median 4 sessions), with birds learning the task after 30 days of training. Out of 36 birds that began training, 9 passed the learning criterion to move on to testing. These 9 birds were unevenly distributed across treatment groups, with 3 birds from 2 low complexity/high density [LC/HD] pens, 3 birds from 2 high complexity/low density [HC/LD] pens, 2 birds from 2 high complexity/high density [HC/HD] pens, and 1 bird from 1 low complexity/low density [LC/LD] pen. Birds from LC/HD, HC/LD, HC/HD, and LC/LD pens learned the task after a mean of 6, 7, 8, and 10 training sessions, respectively.

Testing round tended to impact latencies to approach all cue types (F_1,3_ = 2.35; *p* = 0.074). Latency to approach (s) all cues was 32.96 ± 3.14 s in test round 1, 40.43 ± 3.16 s in test round 2, 36.62 ± 3.14 s in test round 3, and 39.69 ± 3.16 s in test round 4. There was no effect of reward side (left or right) or color (black or white) on latencies to approach during the judgement bias test (F_1,7_ = 3.42; *p* = 0.107).

**Figure 1:**
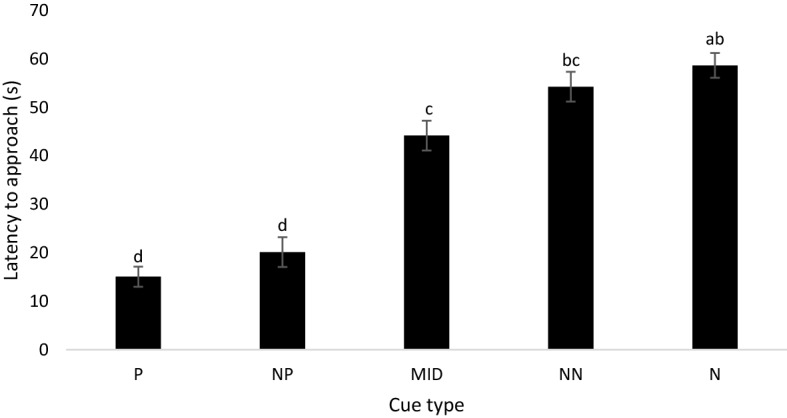
Least squares mean estimates (± SEM) for latency to approach (s) all five cues (positive [P], near positive [NP], middle [MID], near neutral [NN], and neutral [N]) in the judgement bias test for 4 test rounds (n = 9). Means with different superscripts (^a–d^) differ at *p* < 0.001.

**Figure 2:**
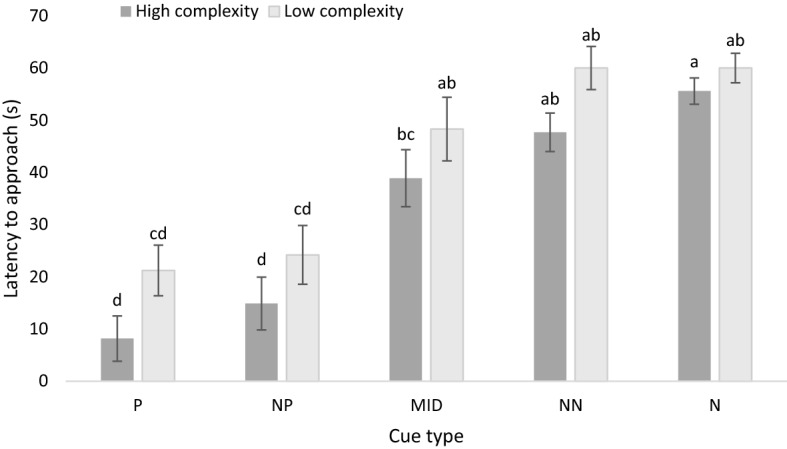
Mean (± SEM) latency to approach (s) all five cues (positive, P; near positive NP; middle, MID; near neutral, NN; and neutral, N) for birds from both high- and low-complexity pens in the judgement bias test for 4 rounds (n = 9). Means with different superscripts (^a–d^) differ at *p* < 0.01.

Across environmental complexity and stocking density treatment groups, birds approached the P (15.08 ± 2.08 s) and NP (20.13 ± 3.07 s) cues faster than the MID (44.19 ± 3.07 s), NN (54.27 ± 3.07 s), and N cue types (58.67 ± 2.57 s; F_2,239_ = 73.64; *p* < 0.001; Fig. 1). Birds from high-complexity pens had a shorter mean latency to approach all cues than birds from low-complexity pens (F_1,6_ = 16.816, *p* = 0.006). Additionally, birds from high-complexity pens approached the NN cue faster than birds from low-complexity pens (F_1,35_ = 4.934, *p* = 0.033; Fig. 2), but no differences between enrichment treatments were found for the P (F_1,89_ = 4.03; *p* = 0.085), NP (F_1,35_ = 1.508, *p* = 0.268), MID (F_1,35_ = 1.325, *p* = 0.261), or N cues (F_1,53_ = 1.35; *p* = 0.283). Birds from high-density pens showed a trend to approach cue types faster than birds from low-density pens (F_1,6_ = 5.767, *p* = 0.053; Table 1). Pairwise differences in latency to approach cues were not found for the density treatment. No interaction effect of enrichment and stocking density was found on latency to approach cue types.

**Table 1:** Mean (± SEM) latency to approach (s) all cues (positive, P; near positive, NP; middle, MID; near neutral, NN; and neutral, N) of birds from both high- and low-density pens in the judgement bias test for 4 rounds (n = 9).

Cue type	Stocking density	
	High^1^	Low^2^
	Mean latency ± SEM (s)	Mean latency ± SEM (s)
P	11.21 ± 5.22	17.41 ± 5.83
NP	17.57 ± 5.61	20.83 ± 6.27
MID	40.03 ± 5.39	46.89 ± 6.02
NN	53.04 ± 4.04	53.31 ± 4.52
N	59.05 ± 2.62	55.69 ± 2.93

^1^42.08 kg/m^2^ at day 50.

^2^23.83 kg/m^2^ at day 50.

### Gait

**Figure 3:**
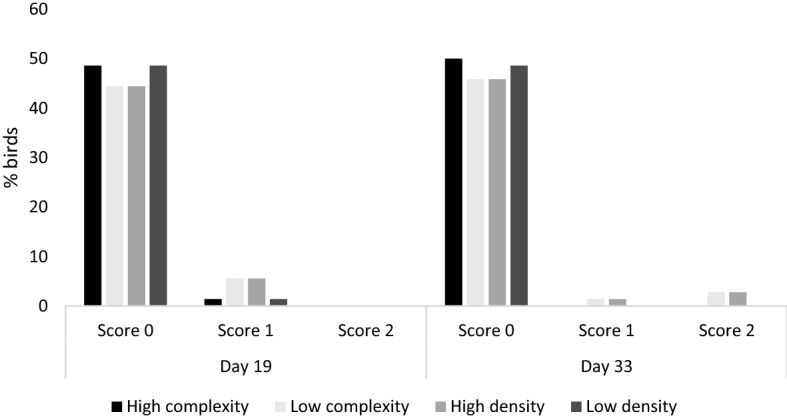
Percentage of birds with each gait score (ranging from 0, walks with no obvious impairments, to 2, unable to walk) in each treatment group. Percentage of birds (n = 72) in high-complexity, low-complexity, high stocking density (42.08 kg/m^2^ at day 50), and low stocking density (23.83 kg/m^2^ at day 50) pens receiving a gait score of either 0, 1, or 2.

No effects of enrichment or stocking density treatments were found on bird gait score (*p* > 0.1). Mean gait scores were 0.125 ± 0.045 for high-density, 0.014 ± 0.014 for low-density, 0.014 ± 0.014 for high-complexity, and 0.125 ± 0.048 for low-complexity pens. Age tended to affect gait scores, with 93.1% of birds receiving a score 0, 6.9% a score 1, and 0% a score 2 on day 19, compared to 95.8% receiving a score 0, 1.39% a score 1, and 2.78% a score 2 on day 33 (F_1,33_ = 2.983, *p* = 0.094; Fig. 3). Gait score was not associated with latency to approach during the judgement bias test (χ^2^ = 0.982, *p* = 0.621).

## Discussion

This study is the first to apply a judgement bias test to evaluate affective state of broiler chickens housed in varying environmental conditions. We attempted to manipulate affective state by placing birds in either high- or low-complexity pens under either a high or low stocking density. Birds were trained to discriminate between multimodal cues, with one color and location associated with a mealworm reward and the opposite color and location unreinforced. Within a relatively limited time frame (approximately 5 weeks), 25% of the selected birds successfully learned to discriminate between the P and N cues. All birds approached the P and NP cues quicker than the MID, NN, and N cues. Birds from HC pens were faster to approach all cue types than birds from LC pens and had a shorter latency to approach the NN cue than birds from LC. Additionally, birds from HD pens tended to approach all cue types faster than birds from LD pens.

In this study, we confirmed that a judgement bias test is an appropriate welfare indicator for broilers. During testing, birds showed longer latencies to approach cues closer to the N cue and shorter latencies for cues closer to the P cue (a generalization gradient^40,70^). The generalization gradient of bird responses shows that broiler chickens can learn a discrimination task and that the judgement bias test is a valid tool to assess affective states in broiler chickens. One methodological issue with judgement bias tests is that subjects can learn that ambiguous cues are unreinforced, causing a reduction in responsiveness that gives the appearance of increasing pessimism despite no change in affective state^40,75^. In line with this, test round showed a trend to impact latency to approach cue types, although latencies did not show a linear increase over time.

Enrichment that allows broilers to express highly-motivated, natural behaviors improves their welfare^40,76,77^. Lower stocking densities compared to commercial standards enables broilers to perform more species-specific behaviors associated with environmental enrichment^78^. In line with our hypothesis, birds from high-complexity pens showed shorter latencies to approach all cues compared to birds from low complexity pens. This faster approach suggests that birds from the complex environment had a greater expectancy to receive a reward compared to birds from the barren environment. Additionally, birds from complex pens approached the NN cue faster than birds from barren pens (12 s difference). This interaction between enrichment and cue type suggests that birds from complex pens were more optimistic than birds from barren pens. However, approaches did not differ for the two other ambiguous cues (NP and MID), suggesting some association between enrichment and affective state (optimism). Our findings are similar to laying hen responses^74^, where ‘exploratory’ layer hens housed with preferred enrichments flipped an ambiguous cue lid more often than exploratory hens housed without enrichments (81% vs. 55% of ambiguous lids, respectively). Although the task was different for broilers in our study compared to their layer hens, both studies suggest that environmental enrichment positively impacts affective states (optimism).

Birds from high-density pens tended to be faster to approach all cues compared to birds from low-density pens. This result was opposite to our predictions, as previous broiler studies have found that high stocking densities lead to fear^79^ and stress^80^, reduced ability to perform species-specific behaviors such as perching^10^, and a higher rate of disturbances of resting birds and aggression in open areas^10,81^. Our results may be attributed to birds housed at high densities having to compete for resources within their home pens, resulting in an increased motivation to receive a food reward during judgement bias training and testing. In support of this explanation, broilers housed at high rather than low stocking densities showed decreased body weights^82^, growth performance^80^, and feed intake^83,84^, possibly due to limited access to feeders^85^. Future research might overcome this limitation by implementing a judgement bias test that does not use feed as a reinforcer, but instead visual cues^62^.

One limitation of our study is that only 9 out of 36 subjects that began training learned the judgement bias task. This may be because our paradigm required birds to walk towards a cue. Broiler chickens are prone to impaired gait, difficulty walking, and lameness as they reach slaughter weight and age^86–89^. This could have affected our subjects’ ability to meet the training criterion, resulting in a low sample size for testing. This explanation is supported by our results showing that gait score increased (worsened) across the study, indicating a worse gait on day 33 than day 19 (Fig. 3). On either day, however, most birds had low gait scores (95.8% receiving score 0 on day 33), suggesting that gait did not impaired birds’ ability to approach cues during judgement bias testing. It is nonetheless possible that even birds with low gait scores could have struggled to walk due to body weight or size. This is supported by results from Bokkers & Koene^90,91^, where fast-growing broilers with low gait scores (good gait) were slower to reach the end of a runway compared to slow-growing broilers at 12 weeks of age (150 s versus 70 s). Training methods need to be refined in order to increase the total number of birds successfully passing the learning criterion. Altering the testing methodology to a pecking task could be a possible solution to these limitations, as evaluated in laying hens^70^. Broilers could be trained to peck at a cue within close proximity, therefore removing the need for locomotion, but still retaining the discrimination response (latency to peck).

This study validated judgement bias tests as a novel approach to assess affective state in broiler chickens, with environmental enrichment increasing optimism consistent with a beneficial effect of enrichment on broiler affective state. Although 75% of birds did not learn the task within 30 days, we demonstrated optimistic responses of the 9 learned birds due to the high-complexity treatment. Thus, judgement bias tests are a promising indicator of affective state in broilers.

## Materials and methods

### Ethics

This experiment was approved by Virginia Tech’s Institutional Animal Care and Use Committee (approval number: 19-175), and animal welfare as prioritized throughout. All methods were carried out in accordance with relevant guidelines and regulations.

### Animals and housing

We conducted this experiment at Virginia Tech’s poultry facility during February and March 2020. Male Ross 708 chicks (n = 1620) were obtained at day 0 from a commercial hatchery (PA, USA) where they were vaccinated for Marek’s disease, followed by transportation to the research facility. Upon arrival, chicks were randomly allocated to one of four treatment groups in a 2 × 2 factorial design using environmental complexity and stocking density as factors at pen level. Each treatment group was replicated three times (12 pens total) and randomly distributed in a block design. Pens (14.5 m^2^) contained standard pine shavings as bedding (approximately 10.15 cm depth), four feeders, and three water lines with nipple drinkers. All birds had ad libitum access to water and commercial broiler chicken feed (starter day 0–14, grower day 15–28, and finisher day 29–50). Birds received heat lamps and 24 h light in the first 7 days, followed by a light:dark schedule of 18L:6D thereafter, with a light intensity of approximately 15 lx during light hours. Due to a technical issue, birds received 24 h light for 7 additional days during week 2 of age. House temperature was gradually decreased from 35 °C on day 1 to 21 °C on day 50 by assessing bird comfort. All birds received a therapeutic dose of antibiotics via the water lines from day 33–40 in response to a disease outbreak that resulted in an increased cull and mortality rate due to pathogen exposure.

### Environmental complexity

Six pens provided a high-complexity (HC) environment, while the other six pens provided a low-complexity environment (LC), similar to commercial standards. HC pens contained four functional spaces (Supplementary Fig. S1), including space for ‘feeding’ (approximately 3 m^2^), ‘comfort’ (approximately 3 m^2^), ‘resting’ (approximately 3 m^2^), and ‘exploration’ (approximately 4.3 m^2^). The ‘feeding’, ‘comfort’, and ‘resting’ spaces included a water line with three nipple drinkers. The feeding space contained four feeders and one third of a medium PECKstone™ (Proteka Inc., VILOFLOSS, Germany) broken into smaller pieces. The comfort space contained a wooden-frame dust bath (180 cm × 91 cm × 10 cm) filled with 68 kg of playground sand (QUIKRETE, GA, USA). Sand was raked and partially replaced when deemed necessary. The resting space included three perches (182.9 cm L × 30.5 cm W × 8.5 cm H) modified from LeVan et al.^92^ and Pettit-Riley and Estevez^81^, using 1.91 cm-diameter PVC pipe, which was sprayed with textured black spray paint (Rust-Oleum, IL, USA) to enhance grip while perching (Supplementary Fig. S2). Birds had access to 7.6 cm of horizontal perch space/bird in high stocking density pens and 15.2 cm in low stocking density pens. The exploration space contained a pair of enrichments. Six enrichments (“toys”) were randomly paired into three groups of two, combining a nutritional and occupational enrichment starting on day 2. These enrichments were rotated every three days according to a randomized schedule to maintain variation and novelty (Supplementary Table S1).

The LC pens provided the same structural space as HC pens, but without enrichments. Four feeders and three drinker lines were distributed throughout the pens (Supplementary Fig. S3).

### Stocking density

Six pens (LC n = 3; HC n = 3) were stocked at a high-density (HD) of 42.08 kg/m^2^ at day 50, with 180 chicks/pen on day 0 (Supplementary Table S2). The other six pens (LC n = 3, HC n = 3) were stocked at a low-density (LD) of 23.83 kg/m^2^ at day 50, with 90 chicks/pen on day 0 (Supplementary Table S2).

### Measurements

#### Judgement bias test

On day 2, 36 chicks (n = 3/pen) were wing banded and gently marked on the upper back with black livestock marker (All-Weather Paintstik, LA-CO Industries, Inc., IL, USA). These markings were reapplied as necessary throughout the experiment. The judgement bias test followed a 4-step process, including habituation, phase 1 training, phase 2 training, and testing (See Supplementary information). All steps of the judgement bias test were video recorded (EOS Rebel T7 DSLR Camera, Canon). The test was performed in an arena (plywood 122 cm × 61 cm with rubber interlocking mats as flooring; Supplementary Fig. 4) and conducted between 07:00–13:00 h. On day 10, the arena was shortened to 91 cm to ensure ease of walking to the far end as the birds aged.

Birds were first habituated to the judgement bias arena by a single observer from day 2–11 (Supplementary Fig. S5). The arena contained four arbitrarily-placed cardboard feed flats (5 cm × 5 cm) filled with a reward (dried mealworms) and two empty black and white containers (4 oz; Ziploc®, S.C. Johnson & Son, Inc.). During habituation, 3 birds from the same pen were placed in the arena for 6 rounds (1 round/day), with each round lasting 5 min. For the first round of habituation, birds were placed directly into the arena with the start box door closed, so birds could not access the start box. For the second habituation round, birds were placed into the start box with the door open so that the arena was clearly visible and accessible. During the following four rounds, birds were placed into the start box with the door closed so birds could not see the arena, and then the door was opened immediately after the third chick was placed into the start box so that the arena was clearly visible and accessible. During round 6, feed flats with mealworms were moved to the far end of the arena from the start box and latency to eat was recorded. Birds were considered habituated when they consumed a mealworm during any of the rounds. All chicks consumed a mealworm at least once after round 6.

Phase 1 of training began on day 13 and was performed by two experimenters. Birds were individually conditioned to walk to the far end of the arena from the start box using a rewarded cue (Supplementary Fig. S6). Birds were individually trained to associate a color cue (100% black or white; n = 17, n = 19) and location (right or left, n = 17, n = 19) with a reward (dried mealworms; both cues counterbalanced). Rewarded cues and locations were balanced across treatments. The color cues (12.2 cm W × 25.4 cm L photo paper) were taped on the far wall of the arena at the preassigned rewarded location (left or right). The arena was divided into 15 cm sections numbered 1 (closest to start box) through 6 (furthest from start box) to record distance walked (Supplementary Fig. S4).

Each phase 1 training session lasted 6 min, with the bird allotted 1 min to approach the rewarded container. A plastic black or white container with mealworms was initially placed at section 1 (approx. 15 cm from start box opening) and moved back to section 2 (approx. 30 cm from start box opening) on the following attempt if the bird approached the container at section 1. This continued until the bird successfully approached the container at section 6 within 1 min. After each 1 min attempt, the observer gently picked up the bird and placed it back into the start box to set up the arena for the next attempt. Unsuccessful attempts were followed by the observer immediately shaking and tilting the container. Birds either would approach the container and eat, after which the container was moved back to a further section, or they would not, after which the attempt was repeated with the container placed at the same section. If the bird did not approach the container after three attempts, the container was moved one section closer to the start box. The observer recorded frequency and latency to approach the container (time from opening start box door until the bird’s head was over the container) and whether the bird ate mealworms (yes/no) from the container. Birds continued to phase 2 of training when they ate mealworms from the container at either all 6 sections consecutively, or at section 6 for all attempts within one session (6 attempts). The first bird reached the learning criterium in session 3 and the last bird in session 10.

Phase 2 of training began on day 23; all training was performed by two experimenters (Supplementary Fig. S7). Positive and neutral cues were presented individually at section 6 according to a pseudorandomized order with no more than two of either cue presented consecutively. Experimenters live-recorded latency (s) to approach cues and whether the bird ate mealworms (yes/no) from the container. Birds continued to the testing stage when they approached the rewarded cue on their own within 1 min 100% of the time that it was presented in a single session and did not approach the neutral cue within a single session consisting of 6 attempts. Inter-observer reliability for all measures of pre-training and training showed a high level of agreement (simple Kappa Coefficient between 0.91 and 1 for approach (1/0), latency to approach (s), eating mealworms (1/0), and distance walked (0–6)).

Depending on when birds met the phase 2 learning criterion, testing occurred from day 39 to day 50 and was performed by a single experimenter, during which the positive (P), neutral (N), and three ambiguous cues (near positive, NP; middle, M; near neutral, N) were individually presented at intermediate locations (75% black/near right, 50% black/middle, and 25% black/near left). Each bird that advanced to testing (n = 9) was tested four times over two days, with 45–60 min in between testing sessions on a single day. Testing sessions lasted a maximum of 7 min, with 1 min attempts for the bird to approach each presented cue (total n = 28 attempts/bird). Cues were presented in a pseudorandomized order. The first and last attempt in a testing session were always rewarded to maintain motivation throughout the test. All other cues were unreinforced. The experimenter live-recorded frequency and latency to approach cues. A ceiling latency score of 60 s was scored for trials when the bird did not approach the cue.

#### Gait

Individual broiler gait score was assessed by a single observer on day 19 and day 33 on 3 birds/pen (n = 36), with the same 36 birds scored on both days. On each gait scoring day, every bird was scored twice (4 observations/bird). The observer entered the birds’ home pen and gently encouraged an individual bird to walk 1.5 m. A plastic PVC pipe was used to herd the subject to a clear path in the home pen and increase distance between the observer and subject. The subject was then given a score out of three categorical descriptors (0–2) from Webster et al.^93^. Score “0” indicated the subject was able to walk at least 1.5 m with no obvious impairment and a balanced gait; score “1” was given to subjects able to walk at least 1.5 m, but showing obvious impairment with a clear limp or awkward gait; and score “2” indicated that the subject was unable to walk 1.5 m, showing severe impairment with or without shuffling on the shanks or hocks with assistance of wings.

### Statistical analysis

Data were analyzed in JMP pro 15 (SAS Institute Inc., Cary, NC, USA). Residuals were deemed normally distributed based on visual inspection of normal quantile plots. For the judgement bias data, we ran general linear mixed effect models with latency as the response variable; enrichment, stocking density, and cue position as fixed effects; and bird ID as a random effect. Interactions between the fixed effects were included in the model and removed if not significant. A Tukey Kramer’s pairwise comparison post-hoc test was used to compare latencies to approach each cue type, with enrichment*cue as fixed effects and bird ID as a random effect. To check for consistency across all four tests for each bird, mixed models were used with test round and cue position as fixed effects, and bird ID as a random effect. General linear mixed effect models were used to test for side bias, with reward side and location as fixed effects, and bird ID as a random effect. Gait data residuals were normally distributed, so these data were also analyzed using general linear mixed effect models; gait score was the response variable, enrichment and density were fixed effects, and bird ID nested within pen were random effects. To test for associations between age and gait score, Pearson’s chi-square test was used with gait score as the dependent variable and day as an independent variable. Wilcoxon rank-sum test was used to test for links between gait score and latency to approach during the judgement bias test, with latency as the dependent variable and gait score as the independent variable. Data are presented as LSmeans ± SEM unless otherwise noted.

## Supplementary Information

Supplementary Information.

## Data Availability

The datasets generated and analyzed during the current study are available from the corresponding author upon reasonable request.
