# Supplementary material for: Environmental complexity positively impacts affective states of broiler chickens
Source: Sci Rep. 2021 Aug 20;11:16966. doi: 10.1038/s41598-021-95280-4 (PMC8379235; doi:10.1038/s41598-021-95280-4)
Supplement: Supplementary file 1 — Supplementary Information. [file 41598_2021_95280_MOESM1_ESM.docx]

**Supplementary information for:**

**Environmental complexity, but not stocking density, impacts affective state of broiler chickens**

Anderson, M.G.^1^, Campbell, A.M.^1^, Crump, A.^2^, Arnott, G.^3^, Jacobs, L.^1*^

^1^Department of Animal and Poultry Sciences, Virginia Polytechnic Institute and State University, Blacksburg, Virginia, USA

^2^Centre for Philosophy of Natural and Social Science, The London School of Economics and Political Science, London, UK

^3^School of Biological Sciences, Queen’s University Belfast, Belfast, UK

^*^Corresponding author: jacobsl@vt.edu


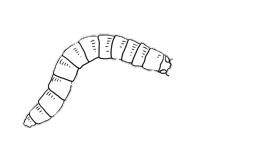


Dust bath

“Play and exploration”

“Feeding”

“Comfort”

“Rest”


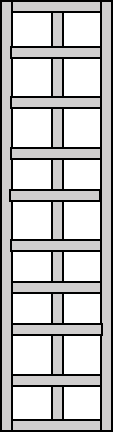

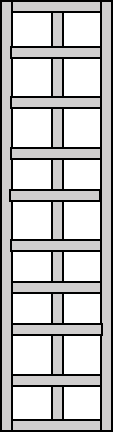

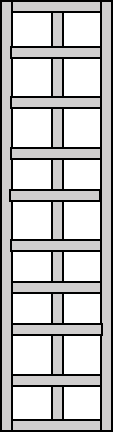


**Figure S1**. Diagram of high-complexity pens (top view). Four functional spaces including ‘feeding’, ‘comfort’, ‘resting’, and ‘exploration’. The feeding space contained four feeders () and pecking stones (not depicted). The ‘comfort’ space included a dust bath (). The ‘resting’ space contained three perches (
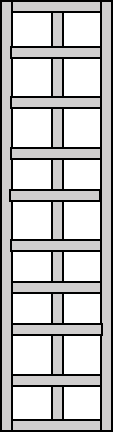
). The ‘exploration’ space contained varying pairs of enrichment objects (not depicted). The ‘feeding’, ‘comfort’, and ‘resting’ spaces each contained a water line with three nipple drinkers ().

8.5cm

182.9cm

30.5cm

**Figure S2.** Diagram of perch design in high-complexity pens. Each high complexity pen contained three perches located in the ‘resting’ space.

**Figure S3.** Diagram of low complexity pens (top view). These pens had a similar set up as the high-complexity pens with four spaces, however, the pens had no specific function. Four feeders () and three water lines (), each with three nipple drinkers, were provided across three of the four spaces.

**P NP MID NN N**

61cm

15cm

6

6

5

5

91cm

4

4

33

33

2

2

1

1

12cm

12cm

**Figure S4.** Top view of the judgement bias arena with distance sections (1-6) and start box (). Birds were placed in the start box prior to a pre-training, training, or testing session. Distance sections were used for placing the reward-associated container during pre-training. Colored cues and associated containers (black or white) were placed at either reinforced location (left or right). During testing, three additional ambiguous-colored cues were placed at intermediate locations (near positive, NP; middle MID; near neutral, NN). Cue position was counterbalanced, so that only half of the birds were trained on this configuration (with P left) and half on an opposite configuration (with P right).

**Figure S5.** Diagram of judgement bias habituation protocol (n=36). Chicks in a pen who consumed mealworms before their pen mates were continually placed in the arena for habituation of remaining chicks.

**Figure S6.** Diagram of judgement bias pre-training protocol (n=36). White boxes contain bird responses and grey boxes contain the next step in pre-training.

**Figure S7.** Diagram of judgement bias training protocol (n=36). White boxes contain bird responses and grey boxes contain the next step in training.

**Table S1.** Pairs of enrichment objects (toys). Description of enrichment objects and schedule of rotation in high-complexity pens (rotated every 3 days).

| Nutritional enrichment | Quantity | Occupational enrichment | Quantity | Start day of rotation (day) | | | | | |
| --- | --- | --- | --- | --- | --- | --- | --- | --- | --- |
| Hanging bundles of white string | 8 | Free-moving metal ball (20.3cm diameter)^5^ filled with alfalfa hay | 4 | 2 | 11 | 20 | 29 | 38 | 47 |
| Yellow treat dispenser (7.6cm diameter)^1^ filled with whole-grain oats | 4 | Colored ball (5.8cm diameter)^2^ | 4 | 5 | 14 | 23 | 32 | 41 |  |
| Laser light^3^ | 5min twice a day | Kong toy (5.6cm diameter)^4^ filled with Iceberg lettuce | 4 | 8 | 17 | 26 | 35 | 44 |  |

^1^Lixit Corp., CA, USA

^2^Click N’ Play, USA

^3^Ethical Products, Inc., NJ, USA

^4^KONG, CO, USA

^5^Darice, OH, USA

**Table S2.** Mean pen stocking densities at day 1, 29, and 50 in kg/m^2^ and birds/m^2^.

| Stocking Density treatment | Day 1 | | Day 29 | | Day 50 | |
| --- | --- | --- | --- | --- | --- | --- |
|  | kg/m^2^ | birds/m^2^ | kg/m^2^ | birds/m^2^ | kg/m^2^ | birds/m^2^ |
| High | 0.52 | 13.85 | 18.93 | 13.14 | 42.08 | 12.31 |
| Low | 0.26 | 6.92 | 9.81 | 6.71 | 23.83 | 6.29 |
